# Synthesis of oleophilic electron-rich phenylhydrazines

**DOI:** 10.3762/bjoc.8.29

**Published:** 2012-02-20

**Authors:** Aleksandra Jankowiak, Piotr Kaszyński

**Affiliations:** 1 Organic Materials Research Group, Department of Chemistry, Vanderbilt University, Nashville, TN 37235; 2 Faculty of Chemistry, University of Lódź, Tamka 12, 91403 Lódź, Poland

**Keywords:** arylhydrazines, methodology, synthesis

## Abstract

Phenylhydrazines **1** substituted with two or three long-chain alkyl, alkoxy or alkylsulfanyl groups were successfully prepared by acid-induced removal of the Boc group in hydrazides **2**. The reaction is carried out with 5 equivalents of TfOH in CF_3_CH_2_OH/CH_2_Cl_2_ at −40 °C for 1.5 min. Under these conditions, the deprotected hydrazine **1** is fully protonated, which increases its stability in the reaction medium. The hydrazines were isolated in 60–86% yields and purities >90%. The hydrazides **2** were obtained in 43–71% yields from aryl bromides **5**, which were lithiated with *t*-BuLi and subsequently reacted with di-*tert*-butyl azodicarboxylate (DTBAD).

## Introduction

Mono-arylhydrazines **I** are important intermediates in the synthesis of a number of heterocycles, including indoles [1] and some azoles (for example [2–3]), many of which exhibit biological activity and are used in drug development [4–6]. Arylhydrazines are also key intermediates in the preparation of stable radicals such as verdazyl [7–9] and benzo[1,2,4]triazinyls [10–12].

The parent phenylhydrazine and many of its electron-deficient derivatives, such as *p*-nitrophenylhydrazine, are stable under ambient conditions and are conveniently obtained by using classical methods, such as the reduction of diazonium salts [13–15]. In contrast, electron-rich arylhydrazines are far less numerous and their preparation is complicated by oxidative instability.

**Figure 1:**
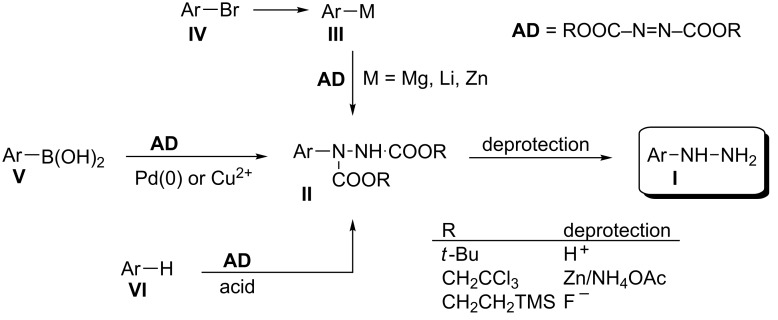
Selected methods for the preparation of arylhydrazines **I** through hydrazides **II**.

To access functionalized and sensitive arylhydrazines several methods involving the deprotection of hydrazides **II** have been developed (Figure 1). Hydrazides **II** are efficiently obtained by the addition of organometallic reagents **III**, prepared from aryl halide **IV**, to azodicarboxylate diesters (**AD**) [16–17]. Alternatively, **II** can be obtained in the Pd(0)- or Cu^2+^-catalyzed reaction of arylboronic acid **V** to **AD** [18–20]. The latter method is especially suited for arylhydrazides substituted with sensitive functional groups. Protected electron-rich arylhydrazines, hydrazides **II**, containing the 2,2,2-trichloroethyl group (R = CH_2_CCl_3_) are conveniently prepared by direct electrophilic amination of arenes **VI** with bis(2,2,2-trichloroethyl) azodicarboxylate (BTCEAD) under Lewis [21–22] or Brønsted [23] acid conditions.

By judicious choice of the substituent R, the removal of the protecting group in **II** and formation of arylhydrazines **I** can be accomplished under acidic (R = *t*-Bu) [16], reductive (R = CH_2_CCl_3_) [24], or nearly neutral (R = CH_2_CH_2_TMS) conditions [22,25]. Among the three methods, the most straightforward is the removal of the Boc group under acidic conditions. Unfortunately, the literature method for deprotection (HCl in isopropanol, 70 °C) has limited scope, and electron-rich 3,4-dimethoxyphenylhydrazine could not be obtained under these conditions, although 4-pentyloxyphenylhydrazine hydrochloride was isolated in 60% yield [16]. The controlled reduction of 2,2,2-trichloroethyl esters (**II**, R = CH_2_CCl_3_) with Zn in aqueous MeOH containing NH_4_OAc gave access to a number of small, electron-rich phenylhydrazines, including 3,4-dimethoxyphenylhydrazine isolated in 76% yield as hydrochloride [24].

**Figure 2:**
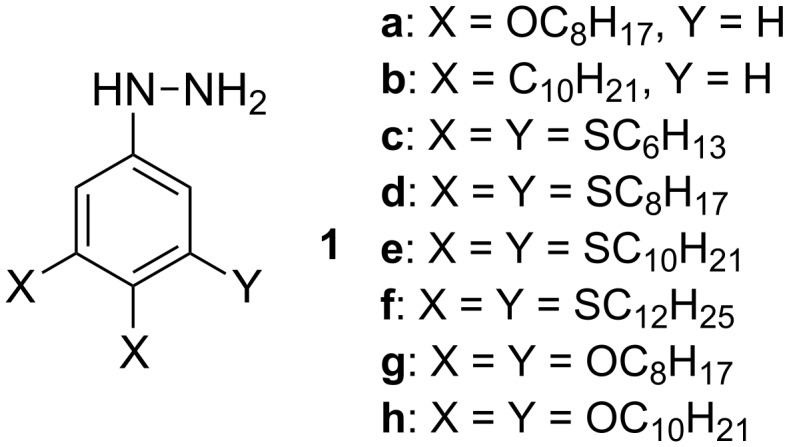
The structures for hydrazines **1a**–**1h**.

In the context of our research program in liquid-crystalline verdazyl derivatives [26], we needed phenylhydrazines **1** (Figure 2) substituted with multiple long-chain alkyl, alkoxy and alkylsulfanyl groups. Here we demonstrate an efficient method for the preparation of several hydrophobic di- and tri-substituted phenylhydrazines in purities sufficient for further chemical transformations. Finally, we demonstrate the application of one of the phenylhydrazines for the preparation of a discotic liquid crystal.

## Results and Discussion

**Scheme 1:**
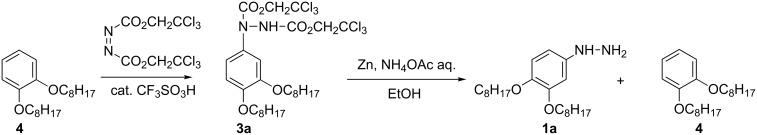
Formation and deprotection of **3a** under reductive conditions.

**Scheme 2:**
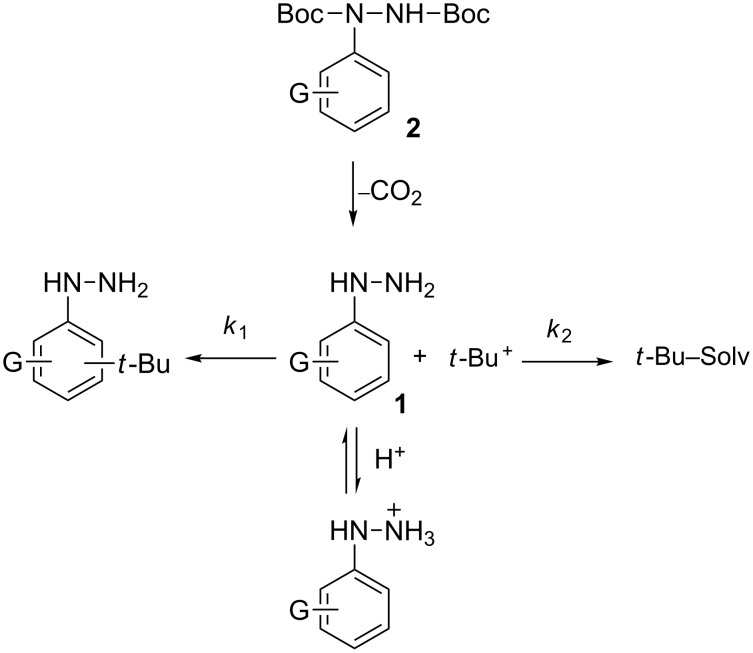
General mechanism for the deprotection of arylhydrazides. G represents a substituent.

Our initial attempts at the preparation of 3,4-dioctyloxyphenylhydrazine (**1a**) focused on deprotection of the trichloroethyl ester **3a** under buffered reductive conditions, according to the general literature procedure [24]. In aqueous MeOH hydrazide **3a** was practically insoluble, and the reaction mixture was triphasic. Under these conditions no formation of hydrazine **1a** was observed. Changing MeOH to EtOH and increasing its volume by two-fold gave homogenous solutions within which the desired hydrazine **1a** was formed along with significant quantities of **4** as the major products (Scheme 1). The deamination product **4** was isolated and identified by comparison with the authentic sample. The yield and proportions of the two products, **1a** and **4**, varied from run to run, according to the ^1^H NMR spectra. Therefore, we focused on the acid-catalyzed deprotection of Boc-substituted hydrazines (Scheme 2), hydrazides **2**, expecting that the reaction could be performed under fully homogenous conditions.

Analysis of the reaction mechanism for the deprotection of **2** under acidic conditions shows that removal of the Boc group generates *t*-Bu^+^, which reacts with the solvent, or alternatively it can alkylate the benzene ring of arylhydrazine (Scheme 2). For less reactive arylhydrazines the former process is faster, *k*_1_ << *k*_2_, and deprotection with HCl in iPrOH is effective [16]. For dialkoxyphenylhydrazines apparently *k*_1_ >> *k*_2_ and the desired hydrazine is not obtained [16].

The nucleophilicity of the hydrazine can be suppressed by its fast and complete protonation with a strong acid (Scheme 2). In this situation, the transient *t*-Bu^+^ is trapped with the solvent, forming volatile products, which simplifies isolation of the hydrazine as a crude product. We have focused on this approach to arylhydrazines employing trifluoromethanesulfonic acid (TfOH), which was used as an effective catalyst in the deprotection of *tert*-butyl aryl ethers [27].

**Figure 3:**
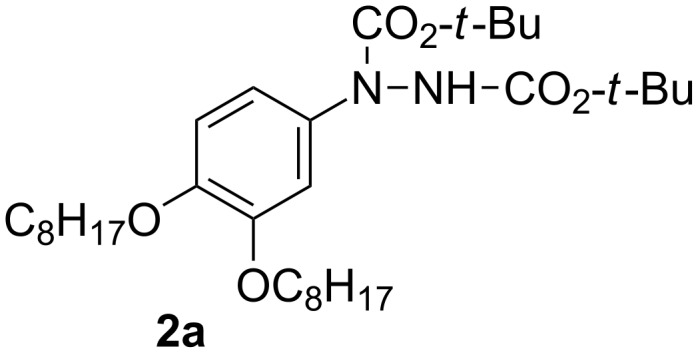
Structure of hydrazide **2a**.

Addition of catalytic amounts of the TfOH acid (10 mol %) to solutions of hydrazide **2a** (Figure 3) in a mixture of CF_3_CH_2_OH/CH_2_Cl_2_ at −40 °C gave little conversion to hydrazine **1a**. With 1.5 equiv of TfOH, hydrazide **2a** was only partially converted to hydrazine **1a**. With 5 equiv of TfOH the reaction was complete in less than 2 min and the crude hydrazine **1a** was isolated as the sole product. Reaction times under 2 min appear to be optimum; the purity of the hydrazine decreased with increasing reaction times.

**Scheme 3:**
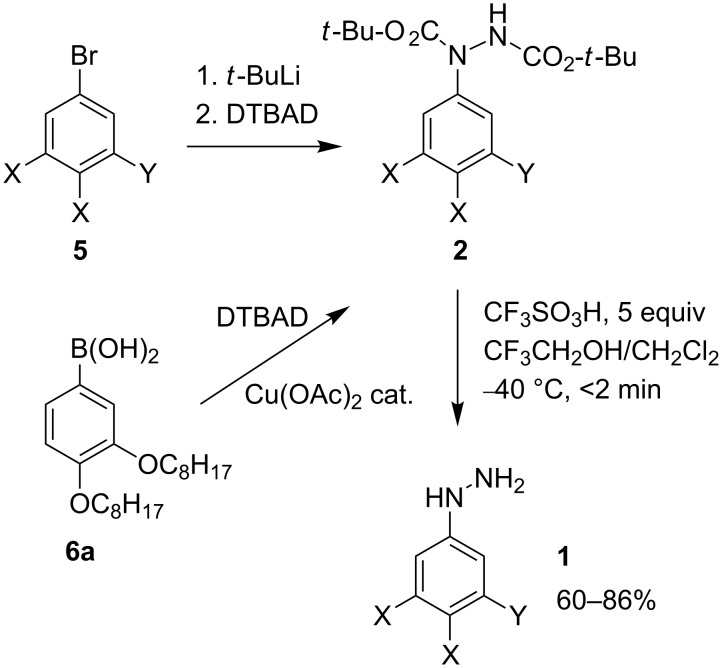
Synthesis of arylhydrazines **1**. Substituents X and Y are defined in Figure 2.

By using this protocol, hydrazines **1** were isolated as viscous oils in purities >90% and yields of 60–86%, according to ^1^H NMR analysis with 1,4-dimethoxybenzene as the internal standard (Scheme 3). Attempts at the preparation of crystalline hydrochlorides of **1** were unsuccessful and the viscous salts rapidly darkened and decomposed.

The Boc-protected arylhydrazines, hydrazides **2**, were conveniently obtained by direct addition of aryllithium to di-*tert*-butyl azodicarboxylate (DTBAD, Scheme 3). The latter was prepared by lithiation of aryl bromides **5** with *t*-BuLi to avoid the formation of *n*-BuBr with *n*-BuLi and N-butylation of hydrazide **2**. Hydrazide **2a** was also obtained by the Cu^2+^-catalyzed addition [18] of arylboronic acid **6a** [28] to DTBAD. The yields of both syntheses of **2a** were comparable.

The trichloroethyl hydrazide **3a** was prepared by acid-catalyzed amination of 1,2-dioctyloxybenzene (**4**) with BTCEAD in the presence of catalytic amounts of TfOH, according to a general literature procedure [23] (Scheme 1).

**Scheme 4:**
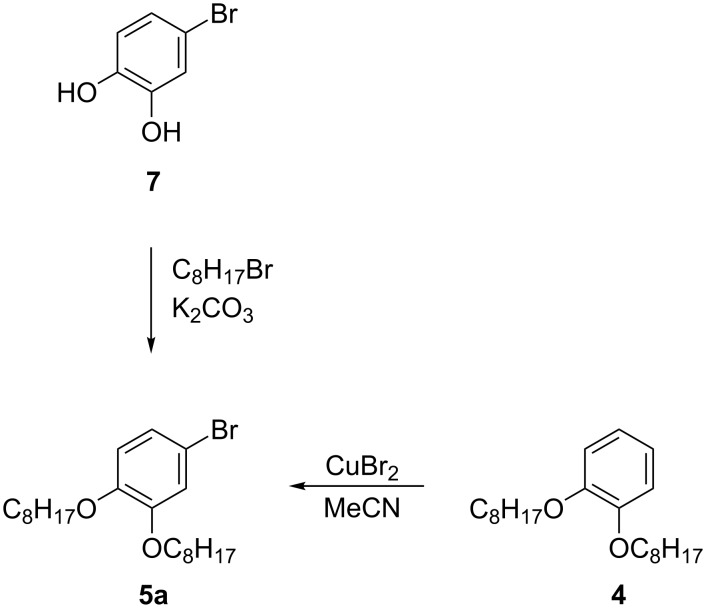
Preparation of bromide **5a**.

The requisite bromobenzene **5a** was prepared by bromination of 1,2-dioctyloxybenzene (**4**) [29] with CuBr_2_ in MeCN according to a literature method [30] (Scheme 4). This method is a convenient alternative to the alkylation of the less readily accessible 4-bromocatechol (**7**) [28].

**Scheme 5:**
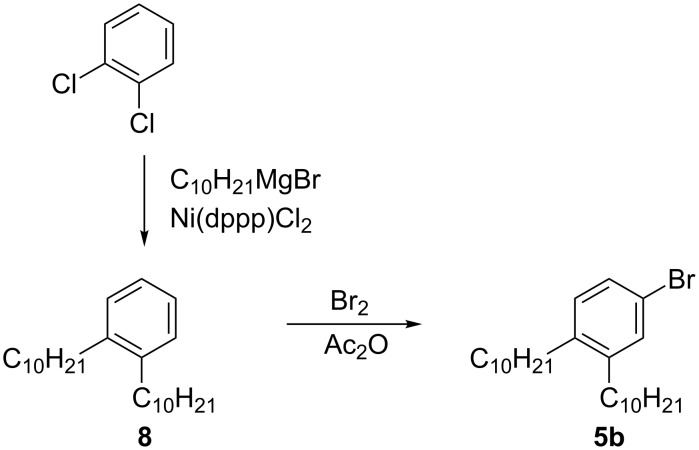
Preparation of **5b**.

1-Bromo-3,4-didecylbenzene (**5b**) was obtained by bromination of 1,2-didecylbenzene (**8**) [31], obtained by the Kumada method [32], with Br_2_ in acetic anhydride (Scheme 5). Typically, the electrophilic bromination of 1,2-dialkylbenzenes results in 4,5-dibromo derivatives as the major products [33–34]. In contrast, the present method permits selective monobromination, although the bromo derivative **5b** was isolated only in about 85% purity. The product could not be purified rigorously from several unidentified contaminants either by chromatography or by distillation due to the lack of separation or partial decomposition. Therefore, crude **5b** was used for the preparation of hydrazide **2b**, which was easily purified by chromatographic methods.

The attempted monoiodination of **8** with BTMA·ICl_2_ by using a general literature method [35] gave only traces of the product and nearly all of the starting material was recovered. Iodination under the Kern conditions [36–37] (HIO_3_/I_2_) gave a mixture of mono- and diiodo derivatives, which were difficult to separate. Manipulation of the reaction time and temperature failed to give the desired monoiodo derivative as the major product.

The preparation of bromobenzenes substituted with alkylsulfanyl groups, **5c**–**5f**, is described elsewhere [38]. Bromides **5g** [39–40] and **5h** [41] were obtained according to the respective literature procedures by alkylation of 5-bromopyrogallol.

**Figure 4:**
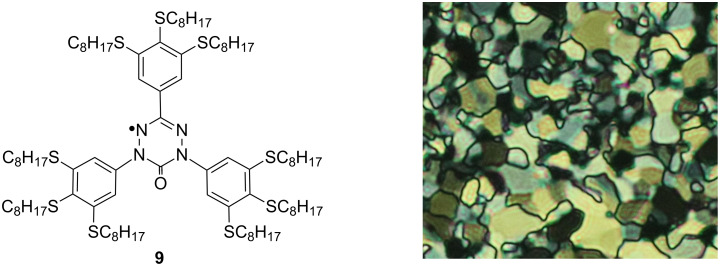
The structure of verdazyl radical **9** and a texture of the *Col*_r_ phase.

The 3,4,5-trialkylsulfanylphenylhydrazines **1c**–**1f** have been used in the preparation of 6-oxoverdazyl derivatives that exhibit liquid-crystalline properties [26]. For instance, radical **9**, prepared from **1d** (Figure 4), exhibits a monotropic columnar rectangular phase (Cr 62 (*Col*_r_ 60) I), a broad absorption band in the visible region, and redox potentials E^0/+1^_1/2_ = +0.99 V and E^0/−1^_1/2_ = −0.45 V versus SCE. Photovoltaic studies of **9** demonstrated hole mobility μ_h_ = 1.52 × 10^−3^ cm^2^ V^−1^s^−1^ in the mesophase with an activation energy *E*_a_ = 0.06 ± 0.01 eV.

## Conclusion

We have developed a synthetic protocol for the efficient preparation of electron-rich phenylhydrazines **1** substituted with alkylsulfanyl, alkyl and alkoxy groups from Boc hydrazides **2**. Experiments demonstrate that the addition of hydrazides **2** to a large excess of TfOH (5 equiv) at −40 °C gives hydrazines **1** in yields ranging from 60–86% and with purity >90%, which is sufficient for subsequent chemical transformations. The optimum reaction time is less than 2 min, typically 90 sec, and longer times lead to a lower purity of the product.

The presented method for the preparation of phenylhydrazines is an attractive alternative to Leblanc’s method, which relies on the reductive deprotection of trichloroethyl hydrazide **3** under heterogenous conditions. Our method involves homogenous solutions, low temperatures and short reaction times, and is particularly suited to oleophilic (“greasy”) arylhydrazines such as **1**, which are important intermediates for the preparation of verdazyls and other heterocycles that may exhibit, e.g., liquid-crystalline properties (e.g., **9**). In comparison with Leblanc’s protocol, our method is also a regiocontrolled hydrazinylation of the aromatics with the more accessible DTBAD through the organolithium. Although we focus on long-chain-substituted phenylhydrazines, we believe that this method can be used for other electron-rich arylhydrazines.

## Experimental

Reagents and solvents were obtained commercially. Reactions were carried out under Ar. ^1^H NMR spectra were obtained at 400 MHz in CDCl_3_ and referenced to the solvent, unless specified otherwise.

### Arylhydrazines **1**

#### General procedure

A solution of hydrazide **2** (1 mmol) in a mixture of CH_2_Cl_2_ (3 mL)/CF_3_CH_2_OH (1 mL) was rapidly added to a solution of TfOH (0.750 g, 0.44 mL, 5 mmol) in CF_3_CH_2_OH (1 mL) at −40 °C under Ar. The mixture was stirred for 1.5 min, and CH_2_Cl_2_ (5 mL) followed by sat. NaHCO_3_ (10 mL) were added under very vigorous stirring. The organic layer was separated and the aqueous layer extracted (3 × CH_2_Cl_2_). Then the extracts were dried (Na_2_SO_4_) and the solvents were evaporated to give crude arylhydrazine **1** in purities typically >90% as a viscous, yellow to orange oil that darkened upon standing. The quantitative analysis of the deprotection reaction was conducted with 0.2 mmol of **2** as described above. The yield of the hydrazines was established by adding known quantities of 1,4-dimethoxybenzene (2.0 mL of 25 mM solution in CH_2_Cl_2_, 0.05 mmol) to the CH_2_Cl_2_ extract, evaporation of the resulting solution, and integration of the low-field ^1^H NMR signals.

**3,4-Dioctyloxyphenylhydrazine (1a):** ^1^H NMR (400 MHz, CDCl_3_) δ 0.88 (t, *J* = 6.8 Hz, 6H), 1.26–1.36 (m, 16H), 1.37–1.47 (m, 4H), 1.70–1.85 (m, 4H), 3.92 (t, *J* = 6.7 Hz, 2H), 3.96 (t, *J* = 6.7 Hz, 2H), 6.34 (dd, *J*_1_ = 8.5 Hz, *J*_2_ = 2.6 Hz, 1H), 6.46 (d, *J* = 2.6 Hz, 1H), 6.82 (d, *J* = 8.5 Hz, 1H); ^1^H NMR (500 MHz, DMSO-*d*_6_) δ 0.86 (t, *J* = 6.7 Hz, 6H), 1.20–1.36 (m, 16H), 1.37–1.46 (m, 4H), 1.61 (quint, *J* = 7.0 Hz, 2H), 1.68 (quint, *J* = 6.9 Hz, 2H), 3.78 (t, *J* = 6.4 Hz, 2H), 3.87 (t, *J* = 6.3 Hz, 2H), 6.24 (dd, *J*_1_ = 8.5 Hz, *J*_2_ = 2.3 Hz, 1H), 6.47 (d, *J* = 2.3 Hz, 1H), 6.71 (d, *J* = 8.6 Hz, 1H).

**3,4-Didecylphenylhydrazine (1b): **^1^H NMR (500 MHz, CDCl_3_) δ 0.88 (t, *J* = 6.9 Hz, 6H), 1.22–1.40 (m, 28H), 1.47–1.58 (m, 4H), 2.51 (t, *J* = 7.0 Hz, 2H), 2.53 (t, *J* = 7.1 Hz, 2H), 2.6 (brs, 3H), 6.59–6.65 (m, 2H), 7.01 (d, *J* = 8.0 Hz, 1H).

**3,4,5-Trihexylsulfanylphenylhydrazine (1c): **^1^H NMR (400 MHz, CDCl_3_) δ 0.87 (t, *J* = 6.9 Hz, 3H), 0.89 (t, *J* = 6.8 Hz, 6H), 1.20–1.35 (m, 12H), 1.36–1.52 (m, 6H), 1.59 (quint, *J* = 7.5 Hz, 2H), 1.71 (quint, *J* = 7.4 Hz, 4H), 2.77 (t, *J* = 7.4 Hz, 2H), 2.83 (t, *J* = 7.3 Hz, 4H), 3.2 (brs, 3H), 6.41 (s, 2H).

**3,4,5-Trioctylsulfanylphenylhydrazine (1d): **^1^H NMR (500 MHz, CDCl_3_) δ 0.87 (t, *J* = 6.9 Hz, 3H), 0.88 (t, *J* = 6.6 Hz, 6H), 1.20–1.34 (m, 24H), 1.38–1.43 (m, 2H), 1.44–1.53 (m, 4H), 1.59 (quint, *J* = 7.5 Hz, 2H), 1.72 (quint, *J* = 7.5 Hz, 4H), 2.77 (t, *J* = 7.5 Hz, 2H), 2.84 (t, *J* = 7.4 Hz, 4H), 6.40 (s, 2H).

**3,4,5-Tridecylsulfanylphenylhydrazine (1e): **^1^H NMR (400 MHz, CDCl_3_) δ 0.87 (t, *J* = 6.8 Hz, 3H), 0.88 (t, *J* = 6.8 Hz, 6H), 1.20–1.35 (m, 36H), 1.36–1.52 (m, 6H), 1.59 (quint, *J* = 7.6 Hz, 2H), 1.71 (quint, *J* = 7.3 Hz, 4H), 2.76 (t, *J* = 7.5 Hz, 2H), 2.83 (t, *J* = 7.3 Hz, 4H), 6.40 (s, 2H).

**3,4,5-Tridodecylsulfanylphenylhydrazine (1f): **^1^H NMR (500 MHz, CDCl_3_) δ 0.88 (t, *J* = 6.8 Hz, 9H), 1.20–1.35 (m, 48H), 1.36–1.51 (m, 6H), 1.59 (quint, *J* = 7.5 Hz, 2H), 1.71 (quint, *J* = 7.4 Hz, 4H), 2.77 (t, *J* = 7.4 Hz, 2H), 2.84 (t, *J* = 7.1 Hz, 4H), 6.40 (s, 2H).

**3,4,5-Trioctyloxyphenylhydrazine (1g):** Soft yellow solid; ^1^H NMR (400 MHz, CDCl_3_) δ 0.88 (t, *J* = 6.7 Hz, 9H), 1.22–1.38 (m, 24H), 1.42–1.53 (m, 6H), 1.72 (quint, *J* = 7.1 Hz, 2H), 1.79 (quint, *J* = 7.1 Hz, 4H), 3.86 (t, *J* = 6.6 Hz, 2H), 3.95 (t, *J* = 6.6 Hz, 4H), 6.06 (s, 2H).

**3,4,5-Tridecyloxyphenylhydrazine (1h):** ^1^H NMR (400 MHz, CDCl_3_) δ 0.88 (t, *J* = 6.8 Hz, 9H), 1.21–1.38 (m, 36H), 1.39–1.64 (m, 6H), 1.65–1.84 (m, 6H), 3.86 (t, *J* = 6.6 Hz, 2H), 3.95 (t, *J* = 6.6 Hz, 4H), 6.06 (s, 2H); ^1^H NMR (400 MHz, C_6_D_6_) δ 0.92 (t, *J* = 6.8 Hz, 9H), 1.22–1.58 (m, 38H), 1.63–1.73 (m, 4H), 1.78 (quint, *J* = 7.1 Hz, 4H), 1.97 (quint, *J* = 8.3 Hz, 2H), 3.89 (t, *J* = 6.4 Hz, 4H), 4.23 (t, *J* = 6.5 Hz, 2H), 6.03 (s, 2H).

### Preparation of hydrazides **2**

#### General procedure

To a solution of the substituted bromobenzene **5** (1.0 mmol) in dry THF (10 mL), *t-*BuLi (1.7 M in pentane, 2.2 mmol) was added under Ar at −78 °C. After 1.5 h a THF (1 mL) solution of di-*tert-*butyl azodicarboxylate (DTBAD, 345 mg, 1.5 mmol) was added dropwise. The mixture was stirred at −78 °C for 0.5 h, then 1 h at rt, and quenched with 5% HCl. The organic products were extracted (Et_2_O), the extracts dried (Na_2_SO_4_), the solvents evaporated, and the residue was passed through a short silica-gel column (hexane/CH_2_Cl_2_ then CH_2_Cl_2_) to give hydrazides **2** as white solids.

**1,2-Bis(*tert-*butoxycarbonyl)-1-(3,4-dioctyloxyphenyl)hydrazine (2a):** Yield 71%; mp 55–57 °C; ^1^H NMR (500 MHz, CDCl_3_) δ 0.88 (t, *J* = 6.9 Hz, 6H), 1.22–1.38 (m, 16H), 1.39–1.51 (m, 4H), 1.49 (s, 18H), 1.73–1.84 (m, 4H), 3.96 (t, *J* = 6.6 Hz, 2H), 3.97 (t, *J* = 6.6 Hz, 2H), 6.71 (brs, 1H), 6.80 (d, *J* = 8.6 Hz, 1H), 6.86–6.92 (m, 1H), 6.93–7.02 (m, 1H); Anal. calcd for C_32_H_56_N_2_O_6_: C, 68.05; H, 9.99; N, 4.96; found: C, 68.35; H, 9.82; N, 5.02.

**Method B:** To a solution of 3,4-dioctyloxyphenylboronic acid (**6a**, 50 mg, 0.13 mmol) in THF (2 mL), di-*tert*-butyl azodicarboxylate (DTBAD, 30 mg, 0.13 mmol) was added followed by Cu(OAc)_2_ (cat) under an Ar atmosphere. The mixture was stirred at rt overnight, the solvent was evaporated and the residue was purified on a short silica-gel column (CH_2_Cl_2_) to give 50 mg (68% of yield) of hydrazide **2a**.

**1,2-Bis(*tert-*butoxycarbonyl)-1-(3,4-didecylphenyl)hydrazine (2b):** Yield 63%; mp 37–38 °C; ^1^H NMR (500 MHz, CDCl_3_) δ 0.88 (t, *J* = 6.8 Hz, 6H), 1.23–1.40 (m, 28H), 1.49 (s, 18H), 1.48–1.59 (m, 4H), 2.52–2.59 (m, 4H), 6.70 (brs, 1H), 7.06 (d, *J* = 8.2 Hz, 1H), 7.08–7.21 (br m, 2H); Anal. calcd for C_36_H_64_N_2_O_4_: C, 73.42; H, 10.95; N, 4.76; found: C, 73.06; H, 10.88; N, 4.74.

**1,2-Bis(*tert-*butoxycarbonyl)-1-(3,4,5-trihexylsulfanylphenyl)hydrazine (2c):** Yield 43%; mp 74–75 °C; ^1^H NMR (400 MHz, CDCl_3_) δ 0.87 (t, *J* = 6.9 Hz, 3H), 0.88 (t, *J* = 6.9 Hz, 6H), 1.23–1.37 (m, 12H), 1.38–1.52 (m, 6H), 1.51 (s, 18H), 1.60 (quint, *J* = 7.4 Hz, 2H), 1.72 (quint, *J* = 7.2 Hz, 4H), 2.81 (t, *J* = 7.5 Hz, 2H), 2.84 (t, *J* = 7.4 Hz, 4H), 6.69 (brs, 1H), 6.99 (brs, 2H); Anal. calcd for C_34_H_60_N_2_O_4_S_3_: C, 62.15; H, 9.20; N, 4.26; found: C, 62.35; H, 9.34; N, 4.22.

**1,2-Bis(*tert-*butoxycarbonyl)-1-(3,4,5-trioctylsulfanylphenyl)hydrazine (2d):** Yield 55% yield; mp 51–52 ^o^C; ^1^H NMR (400 MHz, CDCl_3_) δ 0.84–0.91 (m, 9H), 1.21–1.35 (m, 24H), 1.36–1.50 (m, 6H), 1.51 (s, 18H), 1.60 (quint, *J* = 7.4 Hz, 2H), 1.72 (quint, *J* = 7.5 Hz, 4H), 2.81 (t, *J* = 7.5 Hz, 4H), 2.83 (t, *J* = 7.4 Hz, 2H), 6.69 (brs, 1H), 6.98 (brs, 2H); the analytically pure sample was obtained by recrystallization (MeCN); Anal. calcd for C_40_H_72_N_2_O_4_S_3_: C, 64.82; H, 9.79; N, 3.78; found: C, 64.92; H, 9.56; N, 3.91.

**1,2-Bis(*tert-*butoxycarbonyl)-1-(3,4,5-tridecylsulfanylphenyl)hydrazine (2e):** Yield 56%; mp 50–51 °C; ^1^H NMR (500 MHz, CDCl_3_) δ 0.87 (t, *J* = 6.8 Hz, 3H), 0.88 (t, *J* = 7.0 Hz, 6H), 1.21–1.36 (m, 38H), 1.37–1.51 (m, 6H), 1.51 (s, 18H), 1.60 (quint, *J* = 7.4 Hz, 2H), 1.71 (quint, *J* = 7.4 Hz, 4H), 2.81 (t, *J* = 7.6 Hz, 2H), 2.83 (t, *J* = 7.4 Hz, 4H), 6.70 (brs, 1H), 6.98 (brs, 2H); Anal. calcd for C_46_H_86_N_2_O_4_S_3_: C, 66.78; H, 10.48; N, 3.39; found: C, 66.75; H, 10.07; N, 3.43.

**1,2-Bis(*tert-*butoxycarbonyl)-1-(3,4,5-tridodecylsulfanylphenyl)hydrazine (2f):** Yield 50%; mp 49–51 °C; ^1^H NMR (500 MHz, CDCl_3_) δ 0.88 (t, *J* = 6.7 Hz, 9H), 1.22–1.36 (m, 50H), 1.35–1.51 (m, 6H), 1.51 (s, 18H), 1.60 (quint, *J* = 7.4 Hz, 2H), 1.71 (quint, *J* = 7.2 Hz, 4H), 2.81 (t, *J* = 7.4 Hz, 2H), 2.83 (t, *J* = 7.2 Hz, 4H), 6.69 (brs, 1H), 6.98 (brs, 2H); Anal. calcd for C_52_H_98_N_2_O_4_S_3_: C, 68.52; H, 10.84; N, 3.07; found: C, 68.82; H, 10.83; N, 3.06.

**1,2-Bis(*tert-*butoxycarbonyl)-1-(3,4,5-trioctyloxyphenyl)hydrazine (2g):** Yield 45%; white crystals (MeCN/EtOAc); mp 73–74 °C; ^1^H NMR (400 MHz, CDCl_3_) δ 0.88 (t, *J* = 6.8 Hz, 9H), 1.23–1.38 (m, 24H), 1.38–1.52 (m, 6H), 1.50 (s, 18H), 1.73 (quint, *J* = 7.4 Hz, 2H), 1.77 (quint, *J* = 6.9 Hz, 4H), 3.92 (t, *J* = 6.8 Hz, 2H), 3.93 (t, *J* = 6.6 Hz, 4H), 6.64 (brs, 2H), 6.68 (brs, 1H); Anal. calcd for C_40_H_72_N_2_O_7_: C, 69.32; H, 10.47; N, 4.04; found: C, 69.61; H, 10.43; N, 3.91.

**1,2-Bis(*tert-*butoxycarbonyl)-1-(3,4,5-tridecyloxyphenyl)hydrazine (2h):** Yield 64%; mp 55–57 °C; ^1^H NMR (400 MHz, CDCl_3_) δ 0.88 (t, *J* = 6.8 Hz, 9H), 1.21–1.38 (m, 38H), 1.41–1.54 (m, 6H), 1.50 (s, 18H), 1.67–1.82 (m, 6H), 3.91 (t, *J* = 6.8 Hz, 2H), 3.93 (t, *J* = 6.6 Hz, 4H), 6.64 (brs, 2H), 6.68 (brs, 1H); Anal. calcd for C_46_H_86_N_2_O_7_: C, 70.91; H, 11.12; N, 3.60; found: C, 71.31; H, 11.08; N, 3.65.

**1,2-Bis(2,2,2-trichloroethoxycarbonyl)-1-(3,4-dioctyloxyphenyl)hydrazine (3a):** To the solution of 1,2-dioctyloxybenzene (**4**, 1.10 g, 3.31 mmol) in dry CH_2_Cl_2_ (20 mL), one drop of CF_3_SO_3_H was added under Ar at −78 °C followed by a solution of bis(2,2,2-trichloroethyl) azodicarboxylate (BTCEAD, 1.50 g, 3.97 mmol) in CH_2_Cl_2_ (3 mL). The reaction mixture was stirred for 20 min, warmed up to rt, stirred for 10 min, and quenched with 25% NH_4_OAc. The organic products were extracted (CH_2_Cl_2_), the extracts dried (Na_2_SO_4_), and the solvent evaporated. The viscous residue was passed through a silica-gel plug (hexane/CH_2_Cl_2_ then CH_2_Cl_2_) to give 1.03 g (36% yield) of the hydrazide **3a** as a viscous oil: ^1^H NMR (400 MHz, CDCl_3_) δ 0.88 (t, *J* = 6.7 Hz, 3H), 0.89 (t, *J* = 7.2 Hz, 3H), 1.24–1.39 (m, 16H), 1.41–1.50 (m, 4H), 1.80 (quint, *J* = 7.0 Hz, 4H), 3.96 (t, *J* = 6.6 Hz, 2H), 3.99 (t, *J* = 6.6 Hz, 2H), 4.82 (s, 4H), 6.83 (d, *J* = 8.6 Hz, 1H), 6.97 (d, *J* = 8.2 Hz, 1H), 7.02 (brs, 1H), 7.39 (brs, 1H); Anal. calcd for C_28_H_42_Cl_6_N_2_O_6_: C, 47.01; H, 5.92; N, 3.92; found: C, 46.27; H, 5.72; N, 3.92.

**1-Bromo-3,4-didecylbenzene (5b):** To a solution of 1,2-didecylbenzene (**8**, 1.00 g, 2.8 mmol) in a mixture of Ac_2_O (3 mL) and CH_2_Cl_2_ (3 mL), Br_2_ (0.30 mL, 5.6 mmol) and catalytic amounts of I_2_ were added. The reaction mixture was stirred overnight at rt, water was added, the organic products were extracted (hexane), the extracts dried (Na_2_SO_4_), and the solvents evaporated. The residue was passed through a silica-gel plug (hexane) to give 1.20 g (~85% yield, based on NMR, contained ~15% of at least two impurities) of 4-bromo-1,2-didecylbenzene (**5b**) as a colorless oil: ^1^H NMR (500 MHz, CDCl_3_) major signals δ 0.88 (t, *J* = 6.8 Hz, 6H), 1.20–1.40 (m, 28H), 1.49–1.58 (m, 4H), 2.53 (t, *J* = 7.4 Hz, 2H), 2.55 (t, *J* = 7.5 Hz, 2H), 6.98 (d, *J* = 8.2 Hz, 1H), 7.22 (dd, *J*_1_ = 8.2 Hz, *J*_2_ = 1.8 Hz, 1H), 7.25–7.27 (m, 1H); ^1^H NMR (400 MHz, CD_2_Cl_2_) major signals δ 0.86 (t, *J* = 6.8 Hz, 6H), 1.20–1.40 (m, 28H), 1.49–1.58 (m, 4H), 2.52 (t, *J* = 7.4 Hz, 2H), 2.55 (t, *J* = 7.5 Hz, 2H), 6.99 (d, *J* = 8.2 Hz, 1H), 7.19 (dd, *J*_1_ = 8.1 Hz, *J*_2_ = 2.2 Hz, 1H), 7.25 (d, *J* = 2.1 Hz, 1H); HRMS-EI (*m*/*z*): [M]^+^ calcd for C_26_H_45_Br, 436.2705; found, 436.2726; since **5b** undergoes partial decomposition during attempted short-path distillation (>260 °C/0.2 mmHg), it was used without further purification for the preparation of **2b**.

**1,2-Didecylbenzene (8):** Following a general procedure [31], a solution of 1,2-dichlorobenzene (10.0 g, 68.0 mmol), Ni(dppp)Cl_2_ (370 mg, 0.68 mmol), and *n*-decylmagnesium bromide (272 mmol) in a dry THF (100 mL) was heated under reflux overnight. The crude product was passed through a silica-gel plug (hexane) and short-path distilled (220–230 °C/0.3 mmHg) to collect 11.4 g (48% yield) of 1,2-didecylbenzene (**8**) as a colorless oil: ^1^H NMR (400 MHz, CDCl_3_) δ 0.88 (t, *J* = 6.8 Hz, 6H), 1.20–1.43 (m, 28H), 1.57 (quint, *J* = 7.7 Hz, 4H), 2.59 (t, *J* = 8.0 Hz, 4H), 7.06–7.16 (m, 4H); HRMS–EI (*m*/*z*): [M]^+^ calcd for C_26_H_46_, 358.3600; found, 358.3583.
